# The Effect of Educational Intervention Based on the Theory of Planned Behavior in Prevention of Smoking in Male Students

**DOI:** 10.1155/bmri/6927817

**Published:** 2025-05-09

**Authors:** Tayebeh Rakhshani, Sajedeh Kamranpoor, Amirhossein Kamyab, Asiyeh Yari, Ali Khani Jeihooni

**Affiliations:** ^1^Nutrition Research Center, Department of Public Health, School of Health, Shiraz University of Medical Sciences, Shiraz, Iran; ^2^Department of Public Health, School of Health, Shiraz University of Medical Sciences, Shiraz, Iran; ^3^Department of Community Medicine, School of Medicine, Fasa University of Medical Sciences, Fasa, Iran; ^4^Student Research Committee, Hormozgan University of Medical Sciences, Bandar Abbas, Iran

**Keywords:** educational models, prevention, smoking, students, theory of planned behavior

## Abstract

**Background:** One primary health issue that has significantly impacted a portion of society's youth is smoking. This study evaluates the influence of the theory of planned behavior (TPB) on male high school students' decision-making regarding smoking.

**Methods:** This experimental study was conducted in 2022 on 300 teenage students in Jahrom, Iran (150 individuals in the experimental group and 150 in the control group). The data collection instrument included demographic information, knowledge, and TPB-related questions (attitude, subjective norms, perceived behavioral control, behavioral intention, and smoking prevention behavior). Questionnaires were completed by both groups before and after the conclusion of the program. The experimental group participated in five 60-min training sessions. Data analysis was performed using SPSS 25, employing descriptive tests such as the independent *t*-test, paired *t*-test, and chi-square test (*p* < 0.05).

**Results:** Prior to education, no distinction was witnessed in the components of the TPB between the groups (*p* > 0.05). Postintervention, the intervention group exhibited an increase in knowledge scores from 10.89 ± 1.21 to 13.49 ± 1.64, attitude scores from 33.85 ± 1.23 to 51.69 ± 4.69, subjective norms from 4.52 ± 1.81 to 6.38 ± 1.86, perceived behavioral control from 18.38 ± 3.32 to 31.13 ± 1.54, behavioral intention from 6.93 ± 2.75 to 8.33 ± 2.87, and behavior from 6.75 ± 2.74 to 8.53 ± 2.68 (*p* < 0.001).

**Conclusion:** This study showed the impact of the TPB-based education on smoking avoidance behavior. Consequently, the program designed to avoid smoking can make advantage of this notion.

## 1. Background

Smoking raises the rate of morbidity and mortality, causes early death, and is a significant and avoidable risk factor for cancer [1]. Worldwide, tobacco smoking brings about vital health risks, and it has claimed the lives of around six million individuals [2]. Every year, more than half a trillion dollars are spent to compensate for the financial losses brought on by the consequences of tobacco usage [3]. There are several uses for tobacco, including chewing, smoking, and hookahs [4]. It has long been known that smoking has its negative effects [5]. Moreover, smoking causes damage to all of the body's organs and is the primary cause of the majority of cancers, including kidney, lung, and cervical cancer [6]. It also increases the risk of heart attacks, strokes, and other cardiovascular diseases [7].

Approximately 1.1 billion individuals worldwide, or one-third of all adults over the age of 15, suffer from a smoking addiction. Of these, five million pass away each year from smoking-related issues they provide [8]. In 2020, 22% of the world population use tobacco [9]. Despite a significant decline in smoking prevalence in recent years, 1.1% of Canadians aged 12–17 and 11.4% of those aged 18–34 reported current cigarette use in 2021 [10]. In 2021, tobacco, cigarette, and hookah use in Iran was 14%, 9.3%, and 4.5%, respectively [11].

Studies show that most smokers started smoking during adolescence [12]. The pattern and trend of smoking in Iran are also very interesting, to the extent that the age of starting to use it during adolescence and between 13 and 18 years old has been reported [13]. According to the World Health Organization, over 24% of 15-year-old students globally smoke [14]. In Iran, however, the prevalence of smoking among teenagers varies significantly, ranging from 2.5% to 17%, depending on differences in study definitions, sample age groups, and research locations [15]. These variations illustrate the growing challenge of addressing smoking among Iranian youths. Studies by Ayatollahi et al. [16] and Mohammadpoorasl et al. [17] further emphasize that smoking prevalence among teenagers in Iran is alarmingly high, underscoring the urgent need for targeted preventative measures.

In 1987, Fishben and Ajzen proposed the theory of planned behavior (TPB) [18]. In this approach, three factors—attitude toward the conduct, subjective norms, and perceived behavioral control—predict the intention to undertake a behavior. Attitude regarding the actions is a behavior's appraisal, whether positive or negative, and is based on two substructures: behavioral beliefs and an assessment of the behavior's outcomes, determining the behavior's attitude [18]. Subjective norms are the imagined social pressure an individual feels to engage in or refrain from engaging in the desired activity. People frequently do actions based on what they believe other people to be thinking, and those in close relationships with them may have an impact on their intention to accept a behavior [18].

According to this theory, a person's subjective norm is the result of their motivation to follow the goal action in spite of these expectations multiplied by their normative views. In this situation, someone feels a lot of pressure to use drugs if they think—rightly or wrongly—that close friends and family support the experimental use of drugs [18]. Tavousi et al. [19] used this theory to forecast teenage substance misuse; they demonstrated that the structures account for 36% of the intention and 28% of the behavior. Mirzaei et al.'s [20] use of this theory also explained the variation of 17.9% of fathers' conduct in preventing drug addiction in their children. Bashirian et al. [21] have employed the TPB to predict substance usage in teenagers, demonstrating that attitudes are behavioral controls over substance use as well as subjective standards. Huang et al. [22] demonstrated attitude, subjective norms, and perceived behavioral control in first-year Taiwanese high school students utilizing the TPB as an addiction prevention program, which was successful.

Nevertheless, a large proportion of earlier research has focused on a limited sample size [23–26], within a narrow age group [23, 25–28], or between in danger groups [24, 25], showing the significance of knowledge [23, 25, 27, 28]. Prior research has produced varying outcomes when examining the impact of the interventions [23, 25], underscoring the need for more comprehensive research. To address these gaps, this study investigates the impact of a TPB-informed educational program on a larger, more diverse sample of adolescents aged 14–19 years—a critical age range where smoking initiation is highly likely. By employing a structured, theory-driven approach that incorporates key psychological and social factors, this research is aimed at providing a clearer understanding of the effectiveness of TPB-based interventions in preventing smoking. This approach offers a more robust framework for addressing the rising trend of teen smoking in Iran, with the potential for broader applicability to similar populations worldwide.

## 2. Methods

This pretest–posttest research with a control group took place in 2022. The statistical population was male students aged 14–19 from Jahrom City, Iran.

Based on the study's objectives and type and referencing other research in this domain, taking into account the assumptions of 5% error and 80% power, the effect size of 45% (mean difference 0.45 and standard deviation 1), and a one-to-one ratio using the following formula, 300 people were estimated to conduct the study:

 $$\begin{array}{c} n=\frac{\left(\left(1+r\right)/r\right)s^{2}{\left(z_{1-\alpha /2}+z_{1-\beta }\right)}^{2}}{{\left(\partial \right)}^{2}}. \end{array}$$

The 300 participants were randomly allocated into experimental and control groups, with 150 individuals in each group. A stratified randomization method was employed to ensure balanced allocation based on key demographic variables such as age and smoking status. Participants were first categorized into strata based on these variables, and random numbers were generated using a computer-based randomization tool to assign individuals to either the experimental or control group. Inclusion criteria specified male students aged 14–19 years who had never smoked or had not smoked in the past year and had not received prior training in smoking prevention. Exclusion criteria included refusal to continue participation in the study or missing more than one training session. Informed consent was obtained from all participants prior to their inclusion in the study.

### 2.1. Information Collection Tools

Heidarnia et al.'s questionnaire served as the basis for data collection [29]. The questionnaire comprised two parts: demographic data and the TPB. The demographic section comprised nine items pertaining to characteristics including maternal education, paternal occupation, and monthly income. The second section of the questionnaire comprised 50 items, categorized into five constructs: knowledge (14 items), attitude (11 items), perceived behavioral control (7 items), subjective norms (8 items), and behavioral intention (10 items). The constructs of knowledge, attitude measurement, and perceived behavioral control utilized a 5-point Likert scale, with responses from totally agree (score of 5) to totally disagree (score of 1). In contrast, the Likert constructs for subjective norms and behavioral intention were limited to two options: yes or no. The total scores for the attitude structure will range from 60 to 12, for subjective norms from 7 to 35, for perceived behavioral control from 5 to 25, and for the behavioral intention structure from 5 to 25. The behavior structure comprised 10 questions designed to assess the prevention of smoking and drug use.

### 2.2. Validity and Reliability

Using the consensus of 12 experts, the content validity was first qualitatively examined, and the suggested items were then used. Next, using the content validity ratio (CVR), quantitative validity was computed. Subsequently, the instrument's reliability was verified and assessed through the application of retest procedures and the measurement of the variables' internal correlation (Cronbach's alpha coefficient).

### 2.3. Intervention

The educational program consisted of five sessions of 60 min according to the constructs of the TPB and based on the educational needs according to the prevention of smoking among students. The first and second sessions included the reason for the tendency to smoke; the physiology of the body and the dependence of the mind and the brain; misconceptions regarding the physiological, psychological, and social repercussions of smoking; individual and social expenses; what constitutes a healthy individual; and the perspectives of family, friends, and society.

The third and fourth sessions focused on developing courage, life skills, and recognizing and handling high-risk situations. In the fifth session, topics included recognizing and managing negative mood, substituting rational and upbeat ideas for negative ones, handling stress, and boosting self-esteem. The fifth session also covered identifying one's shortcomings, phobias, communication issues, and past experiences—both good and bad—as well as possibilities for group encouragement and pertinent feedback. Group discussions, role-playing, and brainstorming are the main methods used in these sessions, which are held in the format of 8–15 individuals in an online classroom. At the conclusion of each session, students receive a summary of the subjects covered and a pamphlet that relates to it. Additionally, the data was gathered 2 months following the training (Table 1).

### 2.4. Statistical Methods

Data analysis was conducted using SPSS Version 25. Descriptive statistics, including frequency and percentage, were used to summarize demographic characteristics and key variables. Analytical tests, such as paired *t*-tests, were applied to compare pre- and postintervention scores within each group, while independent *t*-tests were used to compare differences between the experimental and control groups. A chi-square test was employed to assess the homogeneity of demographic variables between groups. Additionally, statistical models were used to evaluate the effect of the intervention on key outcomes, including knowledge, attitude, subjective norms, perceived behavioral control, behavioral intention, and smoking prevention behavior. Significance was set at *p* < 0.05 for all analyses.

## 3. Results

The findings of the study indicated that no significant differences were observed between the experimental and control groups in terms of demographic characteristics such as age distribution, father's occupation, monthly income, or education level, confirming the homogeneity of the groups at baseline (Table 2). Similarly, no significant differences were detected in baseline smoking-related variables, such as smoking duration, between the two groups (*p* > 0.05).

After the intervention, significant improvements across all constructs of the TPB were observed in the experimental group when compared to their preintervention scores. These improvements included increases in knowledge (from 10.89 ± 1.21 to 13.49 ± 1.64), attitude (from 33.85 ± 1.23 to 51.69 ± 4.69), subjective norms (from 4.52 ± 1.81 to 6.38 ± 1.86), perceived behavioral control (from 18.38 ± 3.32 to 31.13 ± 1.54), behavioral intention (from 6.93 ± 2.75 to 8.33 ± 2.87), and smoking prevention behavior (from 6.75 ± 2.74 to 8.53 ± 2.68) (*p* < 0.001 for all comparisons). In contrast, the control group exhibited minimal changes in these constructs, with differences in postintervention scores that were not statistically significant, as shown in Table 3.

Statistical analyses revealed that the differences in the postintervention scores between the experimental and control groups were significant for all constructs of the TPB (*p* < 0.001). These results suggest that the educational intervention based on the TPB had a substantial impact on improving the psychological and behavioral constructs related to smoking prevention.

When the results were analyzed within the framework of the TPB, the intervention was found to significantly influence behavior-related variables, including attitude, subjective norms, perceived behavioral control, and behavioral intention, even after controlling for knowledge-related variables and their changes. This approach allowed for a more precise understanding of the intervention's impact, demonstrating that the observed changes in smoking prevention behavior were not solely due to increases in knowledge but were also influenced by changes in other core TPB constructs.

Overall, the findings indicate that the TPB-based educational intervention was effective in promoting smoking prevention behavior among male high school students in the experimental group, highlighting the value of using a theory-driven approach to address behavior change in this population.

## 4. Discussion

This study is aimed at assessing the effectiveness of an instructional program based on the TPB in preventing smoking among male students in Jahrom schools, Iran. The findings demonstrated a significant increase in the experimental group's knowledge about smoking after the intervention compared to the control group. Previous studies, such as those by Mohammadkhani and Rezaee [30], have identified that students with lower social skills are more likely to smoke. Similarly, Ramzankhani et al. [31] found that nonsmoking students tend to possess higher levels of knowledge about the risks of smoking. These findings align with evidence showing that a lack of information is associated with higher tobacco use, while greater knowledge is linked to a stronger rejection of smoking [32]. Therefore, education plays a critical role in increasing awareness and dispelling misconceptions about smoking, which is a key step toward reducing smoking behavior. Addressing incorrect beliefs about smoking, such as the illusion that life without smoking is less enjoyable, can make the journey to quitting both more feasible and satisfying.

The intervention also revealed a significant improvement in the participants' perceptions and attitudes toward smoking, as reflected in the TPB constructs. Within the cultural context of the study, smoking is socially disapproved of, particularly among young individuals and students, as it is viewed as a behavior that transgresses societal norms and often serves as a precursor to substance dependency. By acquiring life skills through the educational program, participants demonstrated an enhanced ability to manage peer conflicts, cope constructively with challenges, and regulate their impulses. These findings are consistent with research indicating that improved cognitive coping mechanisms reduce the likelihood of engaging in smoking and substance misuse [33].

A considerable increase in the subjective norms related to smoking was observed in the intervention group compared to the control group. This reflects the positive impact of the program in reducing the social acceptability of smoking among peers. Subjective norms, a key component of the TPB, are influenced by the perceived attitudes of friends, family, and other significant individuals. The intervention helped diminish the perceived social pressure to smoke, as students were less likely to consider smoking as a socially acceptable behavior [20]. These findings are supported by Jafarabadi et al. [34], who emphasized the importance of designing educational interventions to influence the attitudes of peers and family in order to reduce social norms supporting smoking.

The program also demonstrated a strong influence on perceived behavioral control, which refers to an individual's belief in their ability to regulate or avoid smoking. Participants in the experimental group showed significant improvements in their confidence to refrain from smoking compared to the control group. According to the TPB, individuals who perceive greater control over their ability to quit smoking are more likely to successfully do so. This finding highlights the importance of fostering self-control and empowering students with strategies to resist smoking, consistent with prior research [35].

The intervention further resulted in a significant increase in the intention not to smoke among the experimental group. This indicates the effectiveness of the TPB-based approach in reducing students' inclination to smoke. The results suggest that a combination of education and persuasive strategies can effectively influence behavioral intentions, ultimately leading to a reduction in smoking behaviors.

Overall, the study findings align with previous research, such as Mohammadi et al. [36], which demonstrated that TPB-based educational programs improve attitudes, subjective norms, perceived behavioral control, and intentions related to smoking cessation. These findings provide strong evidence that structured, theory-driven interventions can lead to meaningful behavioral changes and contribute to the prevention of smoking among adolescents.

## 5. Conclusion

The findings showed the impact of the TPB-based educational program on smoking avoidance behavior. Consequently, the program designed to avoid smoking can make advantage of this notion.

Enhancing individuals' awareness and sensitizing them to smoking-related problems, as elucidated by the educational approach for smokers, have positively influenced their behavior. The findings indicate that employing education grounded in the TPB, which highlights crucial psychological factors in behavior modification, can enhance smoking knowledge, negative attitudes, subjective norms, perceived behavioral intention and control, and overall smoking prevention behavior. Consequently, when smokers get adequate and accurate knowledge, coupled with a favorable attitude toward the manageability of smoking, they choose to embrace healthier practices.

## Figures and Tables

**Table 1:** Intervention program.

**Session**	**Constructs**	**Educational content**	**Plan**
1 and 2	Knowledge and attitude	Introduction and objectives' examination of misconceptions concerning the physiological, psychological, and social consequences of smoking, as well as individual and societal expenses	Generate a comprehensive list of potential outcomes
3 and 4	Perceived behavioral control, subjective norms	The influence of peers and family on children's sexual health care. Examination of factors promoting smoking behavior, life competencies, and the identification and management of high-risk scenarios	By affecting control ideas and perceived authority. By shaping normative views and the incentive to adhere
5	Behavioral intention, behavior	Behavioral intent was cultivated by shaping attitudes about conduct and mental standards, thus motivating moms to adopt practices and techniques to avert smoking	By affecting attitudes toward the action and subjective norms and by affecting a behavioral intention, which relies on attitude toward the conduct and subjective norms

**Table 2:** Demographic variables of the groups.

**Variable**	**Experimental group (** **N** = 150**)**	**Control group (** **N** = 150**)**	**p**
Age			
14–16	60 (40%)	70 (46.7%)	0.64
17–19	90 (60%)	80 (53.3%)	
Father's occupation			
Employee	68 (45.3%)	88 (58.7%)	0.42
Self-employed	55 (36.6%)	52 (34.6%)	
Unemployed	27 (18%)	10 (6.7%)	
Monthly income			
Good	65 (43.3%)	50 (33.3%)	0.44
Medium	45 (30%)	75 (50%)	
Week	40 (26.7%)	25 (16.7%)	
Education			
Illiterate	35 (23.3%)	27 (18%)	0.86
Elementary school	45 (30%)	23 (15.3%)	
Secondary school	30 (20%)	40 (26.7%)	
High school	40 (26.7%)	60 (40%)	

**Table 3:** Comparative analysis of groups regarding TPB constructs' pre- and posttest scores.

**Construct**	**Group**	**Before intervention**	**After intervention**	**p** ** value**
Knowledge	Experimental	10.89 ± 1.21	13.49 ± 1.64	*p* > 0.001
	Control	10.28 ± 1.60	11.20 ± 1.75	*p* = 0.985
	*p* value	0.637	*p* < 0.001	

Attitude	Experimental	33.85 ± 1.23	51.69 ± 4.69	*p* < 0.001
	Control	33.62 ± 1.14	48.16 ± 3.84	0.761
	*p* value	0.198	*p* < 0.001	

Subjective norms	Experimental	4.52 ± 1.81	6.38 ± 1.86	*p* < 0.001
	Control	5.48 ± 1.66	5.97 ± 1.98	0.181
	*p* value	0.1.89	*p* < 0.001	

Perceived behavioral control	Experimental	18.38 ± 3.32	31.13 ± 1.54	*p* < 0.001
	Control	18.50 ± 3.05	21.80 ± 2.90	0.585
	*p* value	0.213	*p* < 0.001	

Behavioral intention	Experimental	6.93 ± 2.75	8.33 ± 2.87	*p* < 0.001
	Control	6.13 ± 1.85	6.96 ± 1.66	0.073
	*p* value	0.127	*p* < 0.001	

Smoking prevention behaviors	Experimental	6.75 ± 2.74	8.53 ± 2.68	*p* < 0.001
	Control	7.76 ± 1.73	7.40 ± 1.59	0.307
	*p* value	0.089	*p* < 0.001	

## Data Availability

Data is available on request.
